# Long-lived proteins and DNA as candidate predictive biomarkers for tissue associated diseases

**DOI:** 10.1016/j.isci.2024.109642

**Published:** 2024-03-28

**Authors:** Xiaosong Liu, Bozidar Novak, Christian Namendorf, Barbara Steigenberger, Yaoyang Zhang, Christoph W. Turck

**Affiliations:** 1 Interdisciplinary Research Center on Biology and Chemistry, Shanghai Institute of Organic Chemistry, Chinese Academy of Sciences, 100 Haike Road, Shanghai 201210, China; 2 University of Chinese Academy of Sciences, Beijing 100049, China; 3 Max Planck Institute of Psychiatry, Proteomics and Biomarkers, Kraepelinstr. 2-10, 80804 Munich, Germany; 4 Max Planck Institute of Psychiatry, Clinical Laboratory, Core Unit Analytics and Mass Spectrometry, Kraepelinstr. 2-10, 80804 Munich, Germany; 5 Mass Spectrometry Core Facility, Max Planck Institute of Biochemistry, D-82152 Martinsried/Munich, Germany; 6 Key Laboratory of Animal Models and Human Disease Mechanisms of Yunnan Province, and KIZ/CUHK Joint Laboratory of Bioresources and Molecular Research in Common Diseases, Kunming Institute of Zoology, Chinese Academy of Sciences, Kunming 650223, China; 7 National Resource Center for Non-human Primates, and National Research Facility for Phenotypic & Genetic Analysis of Model Animals (Primate Facility), Kunming Institute of Zoology, Chinese Academy of Sciences, Kunming 650107, China

**Keywords:** Disease, Protein, Systems biology, Proteomics

## Abstract

Protein turnover is an important mechanism to maintain proteostasis. Long-lived proteins (LLPs) are vulnerable to lose their function due to time-accumulated damages. In this study we employed *in vivo* stable isotope labeling in mice from birth to postnatal day 89. Quantitative proteomics analysis of ten tissues and plasma identified 2113 LLPs, including widespread and tissue-specific ones. Interestingly, a significant percentage of LLPs was detected in plasma, implying a potential link to age-related cardiovascular diseases. LLPs identified in brains were related to neurodegenerative diseases. In addition, the relative quantification of DNA-derived deoxynucleosides from the same tissues provided information about cellular DNA renewal and showed good correlation with LLPs in the brain. The combined data reveal tissue-specific maps of mouse LLPs that may be involved in pathology due to a low renewal rate and an increased risk of damage. Tissue-derived peripheral LLPs hold promise as biomarkers for aging and age-related diseases.

## Highlights

• Mouse metabolic labeling delineates long-lived proteins (LLPs) in 10 mouse tissues
• Widespread and tissue-specific LLPs
• Peripheral LLPs as biomarker candidates for aging and age-related diseases
• Distinct tissue average cellular DNA renewal

## Graphical abstract

**Figure:**
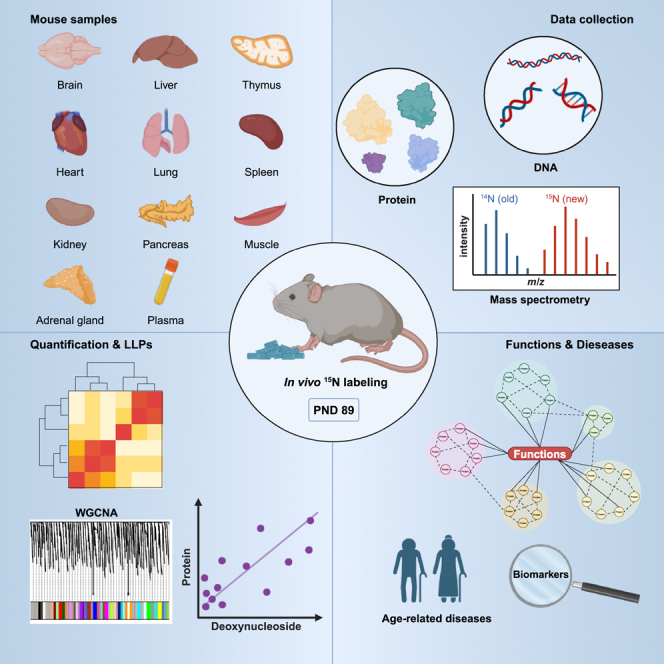

## Introduction

The great majority of cellular proteins is constantly renewed to different extends in order to preserve their integrity and function.^1^^,^^2^^,^^3^ Failure of protein turnover mechanisms is believed to cause cellular dysfunction and pathology associated with several diseases.^4^^,^^5^^,^^6^ For example, a variety of pathological protein aggregates have been found to be involved in different neurodegenerative diseases.^7^^,^^8^ Although proteins are constantly subjected to modification and degradation over the course of their lifetime and therefore need to be replaced by cellular synthesis of new protein, there is a subset of proteins that has very long lifetimes indicating that at least some of the originally translated protein is preserved and can be detected at an advanced age of an organism.^9^^,^^10^^,^^11^ It is highly unlikely that these long-lived protein (LLP) populations, like every other protein, are not affected by misfolding, modification or degradation resulting in their loss of function. The identification of a tissue’s LLPs may therefore provide important information about tissue-associated pathobiology.

In addition to the assessment of LLPs, the determination of long-lived DNA (LLD) in tissues provides a measure for cellular renewal and genome stability. In this regard, it is likely that the extent of a body organ’s renewal depends on its exposure to the environment which in some cases may require continuous replacement.^12^^,^^13^ For example, skin keratinocytes on the surface of an organism are likely to experience mechanical and other damage and therefore need to be frequently regenerated.^13^^,^^14^ On the other hand, tissues consisting of postmitotic cells in protected areas of the body are in general less vulnerable to environmental damage and hence will have a longer lifetime. Human neuronal cells, for example, have been shown to not turn over after birth when development of the central nervous system is completed.^15^ Chromosomal DNA synthesis is not only taking place in dividing cells, but also extensively observed in non-dividing cells.^16^^,^^17^^,^^18^ In this regard, new DNA synthesis in resting cells may be the consequence of incurred DNA damage that requires repair. DNA renewal may thus constitute a biomarker for cell mutagenesis, carcinogenesis, and the aging process. Similar to protein turnover data, DNA renewal data may provide information on tissue-specific disease mechanisms.

Several studies on protein turnover have been carried out that have revealed differences between tissues in rodents and flies.^6^^,^^10^^,^^19^^,^^20^ Similarly, a number of studies have characterized LLPs from different organs and species.^21^^,^^22^^,^^23^^,^^24^^,^^25^^,^^26^^,^^27^^,^^28^ However, most of these studies were focused on specific tissues, such as brain and muscle, revealing nuclear pore and mitochondrial LLPs, respectively. Moreover, LLPs in peripheral body fluids such as blood are yet to be examined in detail. The blood circulatory system provides nutrients and oxygen to body cells, and removes waste products including proteins from organs.^29^^,^^30^ The decline of the circulatory system with aging, is at least in part due to an accumulation of waste proteins, and has been associated with a number of human diseases.^31^^,^^32^ Clearance of macromolecular waste by enzymes is critical to maintain circulatory health. The identification of LLPs in the circulatory system that have not been completely removed may thus provide important information on disease pathobiology.

In this study, we systematically identified LLPs in 10 mouse tissues and the periphery from the same animals using mass spectrometry deep profiling of stable isotope labeled mice. Our data delineate both common and tissue-specific LLPs, including brain LLPs related to neurodegenerative diseases and highlight the accumulation of LLPs in blood. In addition, we have used deoxynucleoside stable isotope incorporation as a proxy for bulk LLD which we compared with LLPs’ stable isotope incorporation in the different tissues. The acquired data suggest that the average renewal of tissue DNA is correlated with that of proteins, albeit at a much lower rate. Due to the increased vulnerability of proteins caused by misfolding, modification, or degradation over time, we submit that LLPs in combination with LLD may add important information on a tissue’s susceptibility toward developing disease.

## Results

### Determination of LLPs and LLD

**Figure 1:**
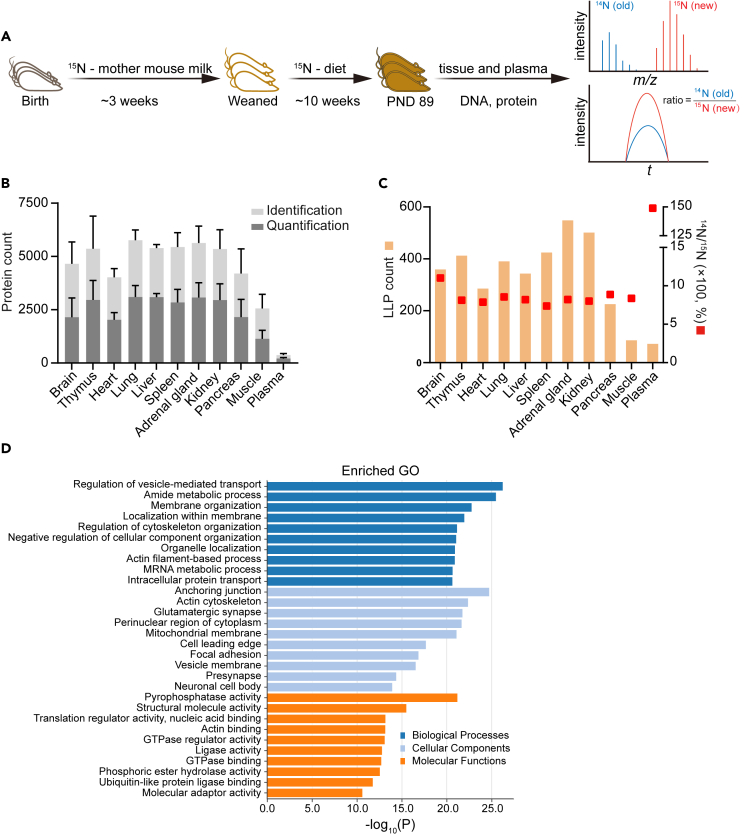
Long-lived proteins in mice (A) Experimental workflow used for the identification of long-lived proteins (LLPs) in mice. Mouse pups received ^15^N-label through mother’s milk for about 3 weeks. After weaning, they were fed ^15^N-labeled diet until postnatal day 89. Proteins and DNA samples were extracted from 10 mouse tissues (brain, thymus, heart, lung, liver, spleen, adrenal gland, kidney, pancreas, muscle) and plasma, and processed for mass spectrometry analysis. (B) Total number of non-labeled and ^15^N-labeled proteins that were identified and quantified in tissues and plasma (Table S1). Data are represented as mean ± SD. (C) Number of LLPs and their ^14^N/^15^N labeling ratios. LLPs are defined as proteins with a ^14^N/^15^N ratio greater than 5% in at least three mice for a tissue, and at least two mice for plasma. (D) Functional GO analysis of all LLPs.

For the determination of LLPs and LLD, mouse pups, immediately after birth, were initially exposed to stable isotope labeling through the breast milk of the mother that was fed a ^15^N-diet (Figure 1A). Following weaning, the pups were then fed the same diet until postnatal day (PND) 89. Ten individual tissues (brain, thymus, heart, lung, liver, spleen, adrenal gland, kidney, pancreas, muscle) and plasma from 3 male and 3 female mice were isolated and proteins and DNA were processed for mass spectrometry analyses. Protein tryptic digests were subjected to shotgun mass spectrometry-based deep profiling using three ion exchange chromatographic fractions for each tissue and plasma sample. DNA from the same tissues was isolated, hydrolyzed, and the isolated deoxynucleosides served as proxy for bulk DNA ^15^N incorporation levels determined by targeted mass spectrometry.

### Quantitative LLPs analysis of mouse tissues and plasma

The mass spectrometry data were analyzed using relative ^14^N/^15^N quantification. The fractional abundance (FA) values of “^14^N %”, representing pre-existing proteins, were also calculated (Table S1). The total number of non-labeled and ^15^N-labeled proteins that were identified and quantified in the 10 tissues and plasma is shown in Figure 1B and Table S1. Approximately 5000 proteins were identified and 2500 proteins quantified from individual tissues, excluding plasma where high-abundance proteins and the great dynamic range of proteins only allowed the identification of proteins that are of moderate to high abundance. We defined LLPs as proteins that were identified with a ^14^N/^15^N ratio greater than 5% in at least three mice for tissues, and at least two mice for plasma (Table S1). LLPs represent proteins derived from tissues made during early development of the mice. A quantitative heatmap of the 2113 identified LLPs from all samples is shown in Figure S1A. The number of LLPs and their corresponding median ^14^N/^15^N ratio, which represents a measure for the turnover degree of these LLPs, in each tissue and plasma is shown in Figure 1C. Keratins were excluded and not considered as LLPs in the following analyses, as they frequently appear as contaminants in proteomics experiments. Dozens to hundreds of LLPs were identified from different tissues. The adrenal gland was shown to have the most LLPs (548) and 359 LLPs were detected from brains. Brain LLPs showed the slowest turnover among all tissues, which is in line with the fact that the brain is mostly composed of non-dividing cells. Strikingly, although the number of identified plasma proteins is small, plasma LLPs showed a much higher ^14^N/^15^N ratio (150%) compared to LLPs in all tissues (<10% in most samples with the exception of brain). Although the number of proteins in plasma is limited for the aforementioned reasons, this result suggests that plasma LLPs of moderate to high abundance may enter the circulatory system at an early time point of development and are not degraded efficiently over time. The functional GO analysis of all LLPs suggests their involvement in various cellular components, including the cytoskeleton, synapse, and mitochondria (Figure 1D).

### Widespread mouse LLPs

**Figure 2:**
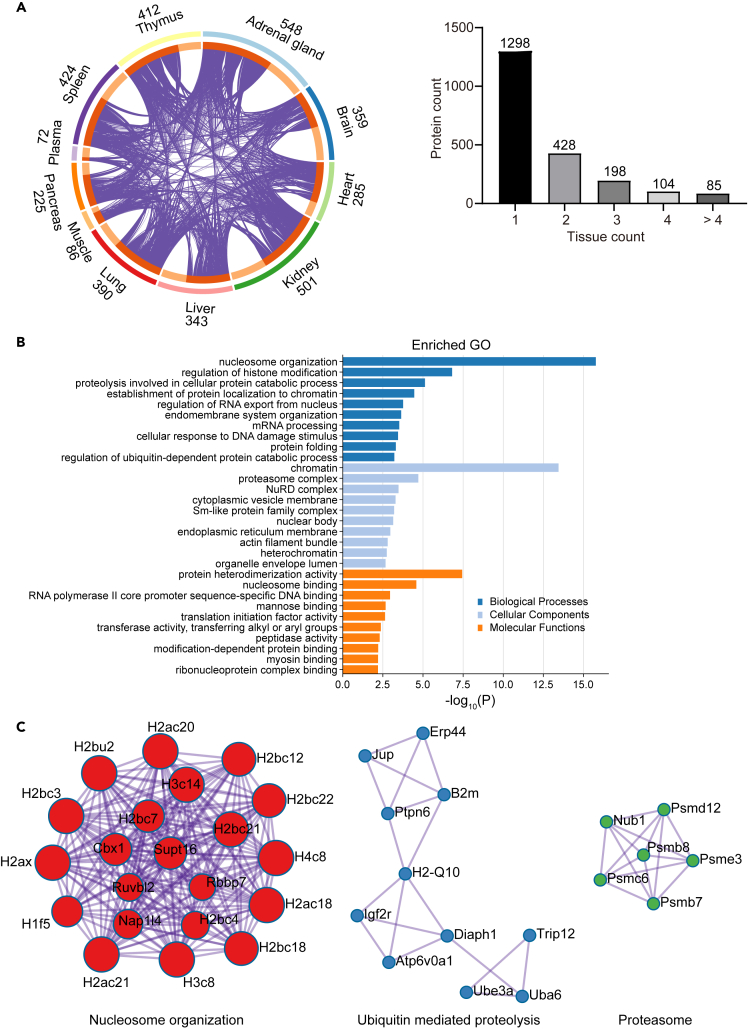
Distribution and functional analysis of widespread long-lived proteins across tissues (A) Long-lived proteins (LLPs) identified across different samples. The circus plot and histogram show the number of LLPs shared between tissues. The 189 LLPs found in at least 4 tissues are defined as widespread LLPs (Table S2). (B) Functional GO analysis of the widespread LLPs. (C) Significant protein-protein interaction networks that are enriched with widespread LLPs. The analyses were performed by Metascape and R.

Next, we investigated which LLPs are commonly represented in different tissues. The circos plot of LLPs tissue distribution (Figure 2A) shows that 1298 out of 2113 LLPs were only detected in one tissue, whereas the remaining 815 LLPs were found in at least two tissues. We classify the 189 LLPs that are shared between more than 3 tissues as widespread LLPs (Table S2). Their functional GO analysis (Figure 2B) and the highlighted protein-protein interaction networks (Figure 2C) suggest that LLPs found in almost all tissues are mainly involved in “chromatin” and “proteasome complex” related components. “Nucleosome organization” proteins including histones and others are part of the nucleolus and hence, like DNA, are well protected from cellular and environmental stress. This may be one reason why these proteins do not undergo high turnover regardless in what tissue, a finding that has also been reported in previous studies.^6^^,^^9^^,^^11^^,^^33^ In addition, we identified LLPs that are part of the “ubiquitin-proteasome system” in all tissues, which is of interest due to its critical function in maintaining proteostasis. The “ubiquitin-proteasome system” is the major machinery for degrading cellular proteins and maintaining functional proteostasis.^34^^,^^35^ Altogether, our data indicate that the nucleosome and “ubiquitin-proteasome system” proteins are inefficiently replenished in multiple tissues.

### Tissue LLPs found in the periphery

**Figure 3:**
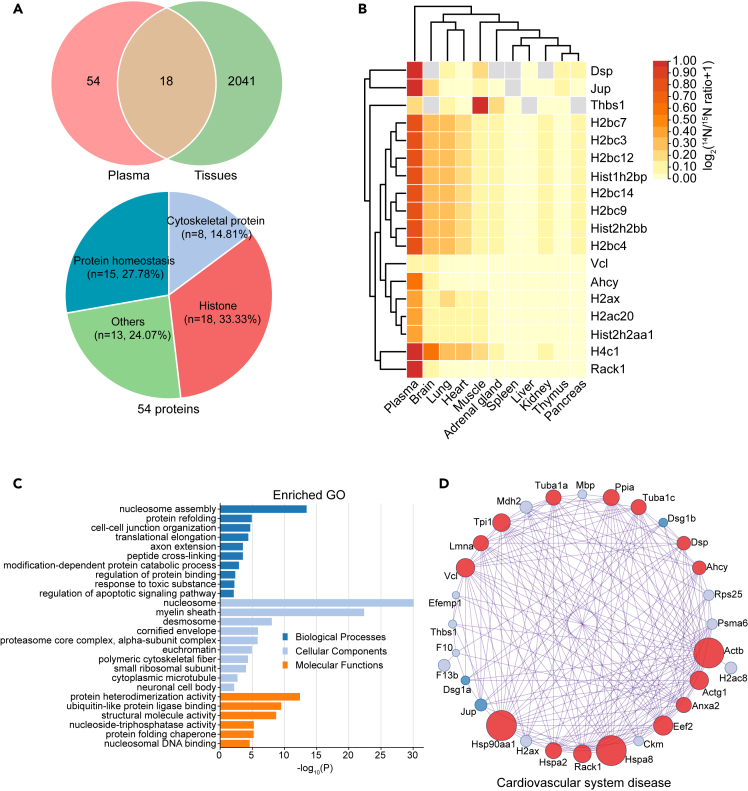
Long-lived proteins in plasma (A) Venn diagram of long-lived proteins (LLPs) that are shared between tissues and plasma. (B) Quantification of the 18 common LLPs between tissues and plasma. The majority of the LLPs identified in plasma are also found in most of the tissues (Table S3). (C) Functional GO analysis of the plasma LLPs. (D) Network of plasma LLPs that are associated with cardiovascular diseases. The analyses were performed by Metascape and R.

Proteins, including damaged proteins that are secreted from cells or leaked after cell death can accumulate in the blood if not removed by the liver and kidney or degraded by proteolytic enzymes.^30^^,^^36^^,^^37^ As we found a significant number of LLPs preserved in plasma, we wondered where these proteins were originated from. Although most of the plasma LLPs were in fact only detected in the plasma (Figure S1B), 18 LLPs were shared with the ones we identified in body tissues (Figure 3A, and Table S3). Interestingly, the 18 shared LLPs had a higher ^14^N/^15^N ratio in plasma than in the tissues they were also found in, including brain, lung, and heart (Figure 3B). At least for this small number of moderate to high abundance plasma LLPs, this suggests that the same LLP either has a much longer half-life in plasma than in the tissue of its origin, indicating an inefficient clearance in plasma, or was made very early during mouse development (“old proteins”), and was continuously extracted into the blood (Figure 3B). The functional analyses using all plasma LLPs classified them into nucleosome, myelin sheath, and desmosome (Figure 3C). Although small in number, it is noteworthy that among the 72 identified plasma LLPs, 30 of them have functional associations with various cardiovascular diseases (Figure 3D). Further studies are required to show that plasma LLPs, in the event of incurring damage over time, may indeed contribute to the onset and progression of cardiovascular diseases.

### Tissue-specific and hub LLPs

**Figure 4:**
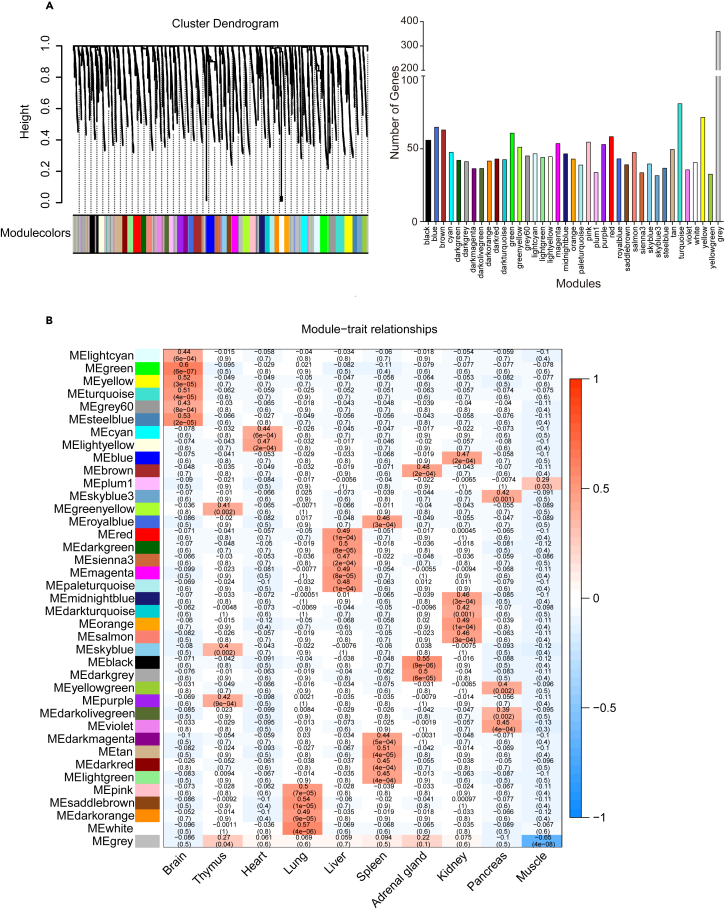
Weighted correlation network analysis (WGCNA) of long-lived proteins across 10 mouse tissues (A) Hierarchical clustering and visualization of gene module partitioning of 2109 genes that encode all identified long-lived proteins (LLPs). Gene number in each of the 39 modules, genes that are not part of any module are in gray. The power value is 8 (Figure S2B). (B) WGCNA specifies tissue-correlated modules and genes. Shown are module-tissue associations. Each row corresponds to a module and each column represents a tissue. Correlations between modules and tissues are displayed with color-coded values. The numbers in each cell represent *p* values (bottom) and correlation value (top).

To explore tissue and gender specific LLPs, a weighted correlation network analysis (WGCNA) was performed (Figures S2A and S2B).^38^ WGCNA clustered all proteins into 39 modules based on the quantitative data (Figure 4A). Each module contains approximately 50 proteins, 400 proteins could not be classified to any module. Following correlation between individual genes or modules (Figures S2D and S2E), the correlation analyses between modules and tissues highlighted their “significant” relationships. For example, brain showed correlation with green (*P* = 6e-07), turquoise (*P* = 4e-05), yellow (*P* = 3e-05), light cyan (*P* = 6e-04), gray60 (*P* = 8e-04), and steel blue (*P* = 2e-05) modules (Figure 4B). Lungs were correlated well with white (*P* = 4e-06), dark orange (*P* = 9e-05), saddle brown (*P* = 1e-05), and pink (*P* = 7e-05) modules (Figure 4B). However, no module had significant association with gender (Figure S2C).

**Figure 5:**
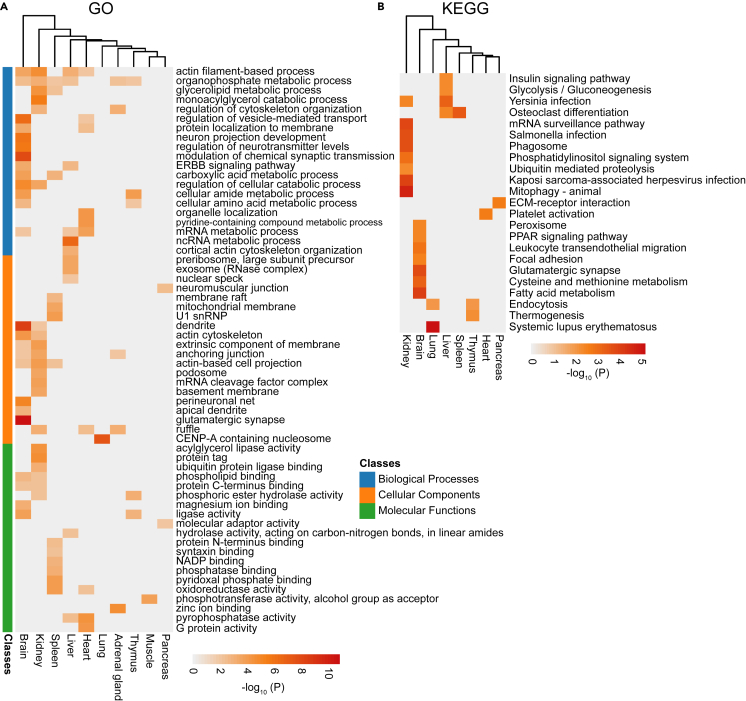
Tissue-enriched modules and hub genes for long-lived proteins Hub genes are defined as those with a gene significance (GS) greater than 0.2 and a module membership (MM) better than 0.8 (Table S4, and Figure S3). (A) Functional GO and (B) KEGG analyses of the tissue-enriched LLPs. The analyses were performed by Metascape and R.

Hub genes that defined a module were further specified. For this purpose, we used “gene”, not “protein”, because the term “gene” is more in line with its customary usage in WGCNA. We defined hub genes as those with a gene significance (GS) greater than 0.2 and a module membership (MM) better than 0.8 in the WGCNA (Table S4, and Figure S3A). Brain has the most, 152, hub genes among all tissues (Figure S3B). GO functions and molecular pathways using tissue-enriched hub genes are shown in Figure 5. Relevant brain functions and pathways enriched with LLPs include “neuron projection development”, “regulation of neurotransmitter levels”, “dendrite”, “perineuronal net”, “apical dendrite”, “glutamatergic synapse”, “modulation of chemical synaptic transmission”, and “glutamatergic synapse” (Figures 5A and 5B). All these pathways are critical for brain physiology and their dysfunction is thought to be involved in neurological and psychiatric disorders.^39^^,^^40^^,^^41^^,^^42^^,^^43^ A number of the same functions and pathways are identified for different tissues.^44^^,^^45^^,^^46^^,^^47^

### LLPs correlating with human diseases

**Figure 6:**
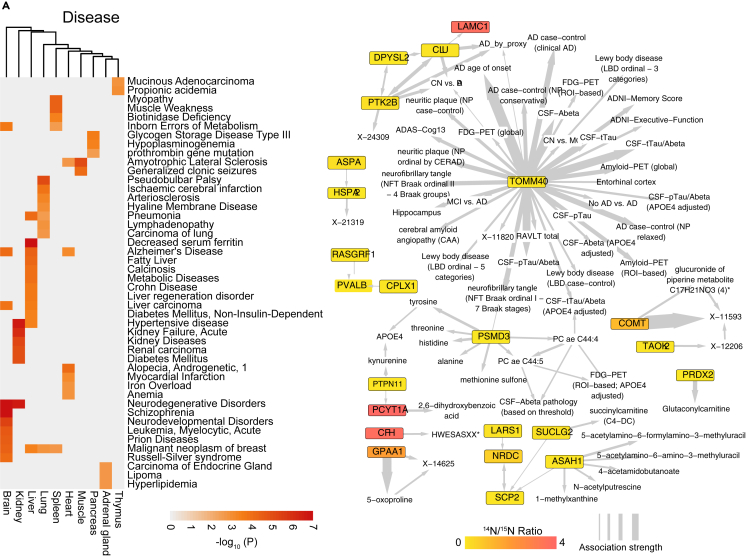
Hub genes and human diseases (A) The association between tissue-enriched long-lived proteins (LLPs) and human diseases. The analyses were performed using DisGeNET database and R. (B) Alzheimer’s Disease-specific LLPs network. ADatlas (https://adatlas.org) was used as the knowledge database to construct the network. Association strength: -log_10_(P).

Several tissue-enriched LLPs are related to diseases that are known to affect the respective tissue (Figure 6A). Due to its low cellular turnover compared to other tissues, the brain is particularly prone to developing LLP-related diseases including neurodegenerative disorders, schizophrenia, neurodevelopmental disorders, and prion diseases (Figures S4A and B). When comparing brain-enriched LLPs to the ADatlas knowledgebase,^48^^,^^49^ a significant number of LLPs was found to be relevant for Alzheimer’s Disease pathology (Figure 6B). In addition, many of those LLPs are predominantly found in the brain (Figure S4C). “Kidney failure”, “acute kidney diseases”, and “renal carcinoma” are all associated with kidney-enriched LLPs. Interestingly, diabetes mellitus, a known risk factor for kidney malfunction, is also enriched with kidney-enriched LLPs. Heart-enriched LLPs are associated with “myocardial infarction”, a common heart disease. In addition, “androgenetic alopecia”, male pattern baldness, which has been reported to be associated with the risk of coronary artery disease^50^^,^^51^ is also associated with heart-enriched LLPs. So is “iron overload”, which has been linked to heart failure.^52^^,^^53^ Several diseases of the liver and lung are also associated with the respective tissue-enriched LLPs, and amyotrophic lateral sclerosis with muscle.

### Average DNA renewal is slower but correlates with protein turnover

In addition to acquiring information on protein turnover, we also analyzed average DNA renewal in the mouse tissues. Mass spectrometry ^14^N and ^15^N signals for bulk DNA-derived deoxyadenosine, deoxyguanosine, deoxycytidine, methyl-deoxycytidine, and thymidine served as a measure for the average cellular renewal in the different tissues at PND 89 (Table S5). They only indicate an average value with regard to longevity and do not reflect the specific longevity of individual DNA molecules in each of the ten tissues which is of relevance for cell cycle and DNA damage repair.

**Figure 7:**
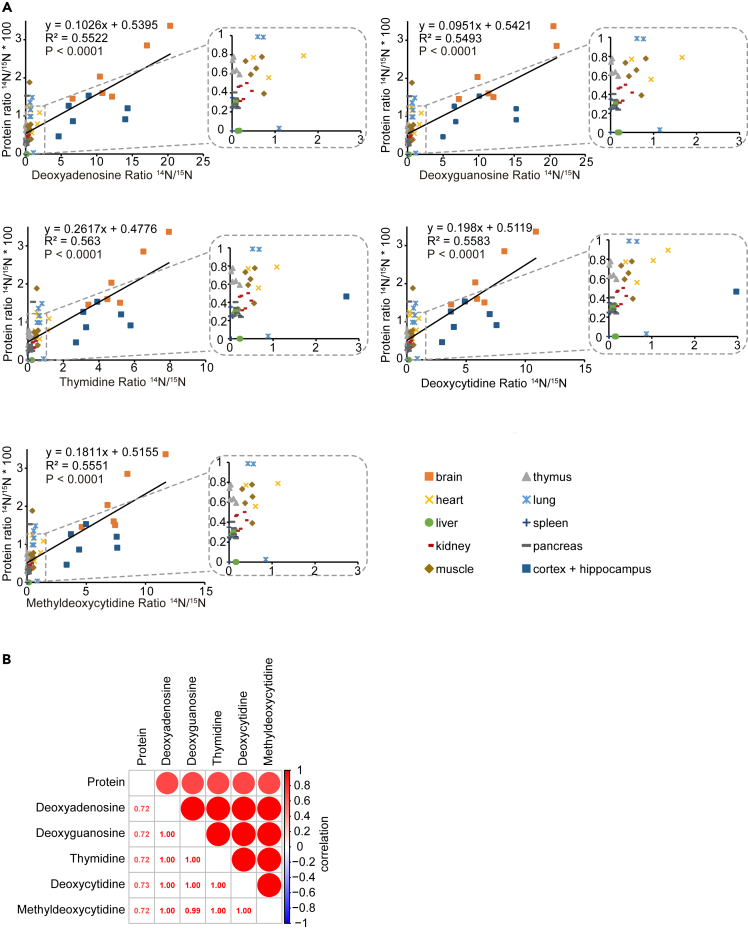
Comparison of ^15^N labeling ratios of long-lived proteins and long-lived DNA (A) Protein and average DNA-derived deoxynucleoside ^14^N/^15^N ratios. Total ^14^N- and ^15^N-peptide spectral counts are used to calculate ratios for proteins. Multiple reaction monitoring mass spectrometry signal intensities are used for deoxynucleosides. (B) Correlation between total protein and DNA-derived deoxynucleosides. The values represent Spearman’s Rank correlation coefficient.

Our data indicate that the average DNA renewal rate is much slower than that of proteins based on the absolute slope values that are all much smaller than 1 (Figure 7A).^54^^,^^55^ This observation is in line with the fact that proteins are spatially and timely more dynamic compared to DNA. After 89 days, by far the most non-labeled DNA is detected in the brain (90.3% ^14^N, Table S5) which is consistent with its low cellular renewal. This is also supported by the highest ^14^N fractional intensities of the identified brain LLPs observed in this (Table S5) and previous studies.^2^^,^^20^ Other tissues vary in their non-labeled DNA amount ranging from a few percent ^14^N signal left in spleen to approximately 46% in the heart (Table S5) which was also reported to be slowly proliferating in another study.^55^ Overall, DNA is on the average significantly more stable than protein as is evident from deoxynucleoside ^14^N signals (Figure 7B).

Purine and pyrimidine nucleobases ^15^N incorporation was similar for all tissues analyzed except for the brain (Figure 7A). Interestingly, we detected approximately twice as much non-labeled deoxyadenosine and deoxyguanosine than deoxycytidine, methyl-deoxycytidine, and thymidine in the brain DNA. Although we were unable to investigate this difference of ^15^N incorporation between pyrimidine and purine nucleobases in the brain in more detail, we can think of two possible reasons for this finding. On one hand, the label incorporation differences may be due to different contributions of *de novo* synthesis and recycling of degraded DNA for the supply of purines and pyrimidines to new DNA synthesis.^56^^,^^57^ In addition, the sources of nitrogen used for *de novo* biosynthesis of purine and pyrimidine are different. Purine nitrogen originates from glycine, glutamine, and aspartic acid, whereas pyrimidines use nitrogen from glutamine and aspartic acid.

Although we only had access to a limited number of ^15^N-labeled cortex tissues, it looks as though that female mice have a greater average cellular renewal in this brain region than males (Table S5) which is consistent with previous reports on rats.^58^ In addition, male mice experienced slower average DNA renewal in heart and lung when compared to female mice (Table S5). The observed smaller ^14^N/^15^N ratios for all deoxynucleosides is consistent with earlier medial prefrontal cortex growth as well as dendrite pruning events in females compared to males and provides an explanation for quicker maturation of female compared to male mice.

In Figure 7 we have compared average ^14^N/^15^N ratios of proteins and DNA-derived deoxynucleosides. Good correlations were found for brain, cortex, and hippocampus. All other tissues have very little unlabeled DNA indicating high cellular renewal. Despite this, LLPs apparently shielded from degradation can still be detected in cells that undergo renewal. The smaller ^14^N/^15^N ratios for thymidine, deoxycytidine, methyldeoxycytidine compared to deoxycytosine and deoxyguanosine may be due to differences in the portion of recycled and newly synthesized pyrimidine and purine deoxynucleosides.

## Discussion

Several studies have been reported on protein turnover in live animals using stable isotopes with a particular focus on the brain because it is the organ with the slowest protein turnover and cellular renewal.^20^^,^^23^^,^^24^^,^^28^^,^^59^ In the current study we have expanded the search to other mouse organs with the aim to identify LLPs that may be of interest for the pathobiology of the affected organ. We realize that mouse body weight and tissue sizes changed from birth to PND 89, potentially masking the identification of LLPs of rapidly growing tissues. However, the criterion used to identify an LLP is based on a ^14^N/^15^N ratio greater than 5%, a relative measure rather than an absolute value, which ensures that a substantial signal from pre-existing protein is obtained for all LLPs. Moreover, we submit that the ^15^N metabolic labeling of all amino acids approach employed in this study is more suitable for *in vivo* LLP studies compared to single-amino acid labeling. Using the latter method, tryptic peptides from partially labeled newly synthesized proteins are frequently observed. As a consequence, the labeled and unlabeled tryptic peptide signals will interfere with tryptic peptide signals from preexisting unlabeled protein.^2^^,^^60^

Our data show the presence of LLPs in all tissues that were analyzed. However, as previously reported for other protein turnover studies, there are differences between tissues when it comes to the fraction of old proteins left at an advanced age of the mouse. Here, the brain stands out as the organ with more LLPs than all other organs. It also is the organ with the smallest degree of cellular renewal as judged by the amount of LLD. In this regard, previous studies with human brain tissue have shown that whereas non-neuronal brain cells are exchanged to some extent,^61^ adult neurogenesis declines with age and correlates with neurological diseases severity.^62^ However, with our data using bulk tissue, we are unable to assess in which cell types the LLPs are most predominantly present. Many LLPs are associated with molecular pathways that are critical for brain homeostasis and have been reported to be involved in brain disorders that have become prevalent in aging societies.^10^^,^^25^^,^^26^^,^^28^^,^^39^ It is reasonable to assume that even a protected organ as the brain becomes vulnerable to the development of disease over time. Proteins are the functional entities of cells and can lose their function due to misfolding, modification, and degradation which ultimately causes disease. The knowledge of proteins that have a significant fraction of old protein may therefore shed light on mechanisms that are involved in disease pathobiology. In the current study we have delineated molecular pathways and tissue diseases that are enriched with LLPs. These include relevant molecular pathways that are important for brain homeostasis and neurologic and psychiatric disorders pathology. We identified LLPs that are part of the “ubiquitin-proteasome system” in all tissues, which is of interest due to its critical function in maintaining proteostasis. The “ubiquitin-proteasome system” is the major machinery for degrading cellular proteins and maintaining functional proteostasis.^34^^,^^35^ A dysfunctional “ubiquitin-proteasome system” caused by inhibited turnover of its component proteins may thus impact its ability to degrade substrate proteins and thereby significantly contribute to global cellular aging and ultimately disease. In this regard, the “ubiquitin-proteasome system” has been implicated in neurodegenerative disorders.^63^^,^^64^^,^^65^^,^^66^

Other tissues also have LLPs that are enriched in functions and pathways that link them to disease such as myocardial infarction (heart), kidney failure (kidney), fatty liver (liver), amyotrophic lateral sclerosis (muscle), and carcinoma of endocrine gland (adrenal gland). A mechanistic explanation for how the respective LLPs are involved in disease mechanisms is beyond the scope of this study. However, we submit that LLPs may provide valuable complementary information to genetic data with regard to disease association.

A particular surprising result of our study is the significant percentage of LLPs of moderate to high abundance identified in plasma. In addition to histones, heat shock proteins, ubiquitin proteins, and other proteosome members, plasma has several cytoskeletal LLPs that are presumably derived from tissues and synthesized during early development. Blood circulation may have an important function in preventing the accumulation of damaged proteins in tissues. Since due to their age, LLPs are particularly prone to incur damage, they are like all other proteins continuously removed from the tissues and found in relatively great numbers in plasma. Although the number of plasma LLPs identified in our study is limited for the aforementioned reasons, the result suggests that plasma LLPs of moderate to high abundance may enter the circulatory system at an early time point of development and are not degraded efficiently over time. Their accumulation to a significant extent during aging may have an impact on the development of cardiovascular diseases, which are the leading cause for death globally.

We also assessed cellular renewal in the tissues using the average non-labeled fraction DNA-derived deoxynucleosides as a proxy. The LLD fraction in a tissue can be a measure of the extent of cellular division. A tissue where most cells are in active cell cycle has a small fraction of LLD, whereas a tissue with more post-mitotic cells will have a greater LLD fraction. Average DNA ^14^N/^15^N ratios assessed in deoxynucleosides are significantly higher than the ones found for LLPs. This is especially the case for the brain and indicates that cellular renewal is far less prevalent than protein renewal. However, overall there is a good correlation between average brain LLD and LLPs ^14^N/^15^N ratios. A recent study by Hasper et al. has also used the ^15^N-SILAM approach to assess protein and DNA renewal in several mouse tissues.^55^ With their method termed “turnover and replication analysis by 15N isotope labeling” (TRAIL), the authors report simultaneous measurements of protein lifetime and cellular lifetime from the same tissue. They find that LLPs have different life cycles in postmitotic versus proliferative tissues. With regard to DNA label incorporation the authors were able to show that ^15^N-diet-derived nucleic acids and *de novo* synthesized nucleic acids were preferentially incorporated into newly synthesized DNA and unlike in our study no difference in label incorporation was found between pyrimidine and purine deoxynucleosides. We speculate that the differences we observed between pyrimidine and purine deoxynucleoside label incorporation are due to the fact that the brain is by far the organ with the slowest cellular renewal, and unlike in other tissues the nucleotide salvage pathway may contribute more significantly to newly synthesized DNA.

Due to its location in the nucleosome, DNA is more protected and less vulnerable toward environmental insults than proteins. Although an intact cell may not undergo renewal, its proteins are always actively turned over as long as the cell is alive. Due to this dynamic nature of the proteins, they represent a better measure for cellular health. A protein with a significant fraction of old protein that has not been turned over will make it more vulnerable toward functional loss. Further studies on LLPs and their incurred molecular alterations as they relate to their functions are therefore warranted to uncover detailed information on disease pathology.

### Limitations of the study

Although our proteomics analysis achieved substantial depth, we note that the detection of some low-abundance LLPs may be obscured during data dependent acquisition shotgun analysis. Furthermore, a protein with an identical absolute half-life may be categorized as an LLP in one tissue but not in another. The LLP definition is determined by relative quantification of labeled and unlabeled signals, which can be influenced by tissue growth and the rate of cell mitosis during the labeling period.

## STAR★Methods

### Key resources table

**Table:** 

REAGENT or RESOURCE	SOURCE	IDENTIFIER
**Chemicals, peptides, and recombinant proteins**		

Iodoacetamide (IAA)	Sigma-Aldrich	Cat#I1149
Urea	Sigma-Aldrich	Cat#U5128
Tris (hydroxymethyl) aminomethane (Tris)	Sigma-Aldrich	Cat#T5941
Dithiothreitol (DTT)	Sigma-Aldrich	Cat#43819
Trypsin	Promega	Cat#V511A
Formic acid (FA)	Sigma-Aldrich	Cat#56302
Triethylammonium bicarbonate (TEAB)	Sigma-Aldrich	Cat#241059

**Critical commercial assays**		

U-15N-labeled SILAM Mouse Diet	Silantes GmbH	Cat#231304650
BCA Protein Assay Kit	Thermo Fisher Scientific	Cat#23225
Albumin IgG removal kit	Merck	Cat#122643
FitAmp General Tissue section DNA Isolation Kit	Epigentek	Cat#P-1003
EpiQuik One-Step DNA Hydrolysis Kit	Epigentek	Cat#P-1023

**Experimental models: Organisms/strains**		

DBA/2Ola mice	in-house	N/A

**Software and algorithms**		

Integrated Proteomics Pipeline (IP2)	Integrated Proteomics Applications	version 4.3.2
Metascape	https://metascape.org/	version 3.5
RStudio	Posit Software, PBC	version 1.4.1717
GraphPad Prism	GraphPad software	version 8.0.2
Ingenuity Pathway Analysis	QIAGEN	version 94302991

**Other**		

30-kD ultrafiltration unit	Merck Millipore	Cat#UFC5030BK

### Resource availability

#### Lead contact

Further information and requests for resources and reagents should be directed to and will be fulfilled by lead contact, Dr. Christoph W. Turck (turck@psych.mpg.de).

#### Materials availability

This study did not generate new unique reagents.

#### Data and code availability

• The raw MS data have been uploaded to iProX with the accession number: IPX0006843000.
• This study does not report the original code.
• Any additional information required to reanalyze the data reported in this study is available from the lead contact upon request.

### Experimental model and study participant details

#### *In vivo*^15^N-labeling

All animal experiments and protocols have been reviewed and approved by the Institutional Animal Care and Use Committee of the Max Planck Institute of Psychiatry. Immediately after birth the DBA/2Ola mother mice were fed with ^15^N-labeled *Ralstonia eutropha* bacterial protein-based rodent diet (Silantes GmbH, Munich, Germany). The same diet was then fed the mouse pups following weaning. The ^15^N-labeled animals did not show any discernible health effects compared to animals fed with a standard diet and had similar weight gains as animals fed with standard food (data not shown). At postnatal day 89 the mice were sacrificed. Blood was taken by cardiac puncture, and plasma was obtained by centrifuging the blood in an EDTA pre-added tube at 1,300 × g for 10 min. The remaining body blood was removed by 0.9% saline perfusion of the heart for 5 min. Subsequently 10 organs (brain, thymus, heart, lung, liver, spleen, adrenal gland, kidney, pancreas, muscle) were collected. Hippocampus and cortex were dissected from four additional mouse brains (Below Table). The organs and plasma were snap-frozen in liquid nitrogen and stored at −80°C for further usage.

**Table:** Summary of analyzed mouse tissue and plasma samples

Mouse ID	Gender	Heart	Kidney	Liver	Lung	Muscle	Pancreas	Brain	Spleen	Thymus	Adrenal gland	Cortex	Hippocampus	Plasma
Mouse # 1	Female	•	•	•	•	•	•	•	•	•	•	┅	┅	┅
Mouse # 2	Female	•	•	•	•	•	•	•	•	•	•	┅	┅	┅
Mouse # 9	Male	•	•	•	•	•	•	•	•	•	•	┅	┅	•
Mouse # 10	Male	•	•	•	•	•	•	•	•	•	•	┅	┅	•
Mouse # 11	Male	┅	┅	┅	┅	┅	┅	┅	┅	┅	┅	•	•	┅
Mouse # 12	Male	•	•	•	•	•	•	•	•	•	•	•	┅	•
Mouse # 16	Female	┅	┅	┅	┅	┅	┅	┅	┅	┅	┅	•	•	┅
Mouse # 18	Female	┅	┅	┅	┅	┅	┅	┅	┅	┅	┅	•	┅	•
Mouse # 19	Female	•	•	•	•	•	•	•	•	•	•	┅	┅	┅

### Method details

#### Tissue protein preparation

Tissues (10–40 mg) were homogenized for 1 min with 200 μl SDS-lysis buffer (2% SDS, 100 mM Tris-HCl, pH 7.6) and a spatula tip of 0.5 mm zirconium beads using a bullet blender (Bertin Technologies, Montigny-le-Bretonneux, France). The samples were then centrifuged at 16,000 x g for 10 min at RT and protein concentration determined with BCA Protein Assay Kit (Thermo Fisher Scientific, Darmstadt, Germany). The Filter Aided Sample Preparation (FASP) II method was used for protein digestion into peptides.^67^ The Microcon YM-30 filter cartridges (Merck, Darmstadt, Germany) were first washed with 100 μl 8 M urea, 50 mM Tris-HCl, pH 8.5. 100 μg protein extract was mixed with 100 μl 8 M urea, 5 mM Tris-HCl, 5 mM DTT, pH 8.5 in the filter unit and incubated for 1 h at 37°C. After centrifugation the retentate was washed three times with 8 M urea, 50 mM Tris-HCl, pH 8.5 before adding 100 μl 8 M urea, 50 mM Tris-HCl, 10 mM iodoacetamide, pH 8.5 and mixing at 400 rpm for 45 min in the dark. After centrifugation the retentate was washed three times with 100 mM triethylammonium bicarbonate (TEAB) (Sigma Aldrich, St. Louis, MO, USA) and then transferred to a new collection tube. 150 μl 100 mM TEAB and 2.5 μg trypsin were added and incubated overnight at 37°C. After centrifugation, 150 μl 100 mM TEAB were added and again centrifuged resulting in 200 μl filtrate containing tryptic peptides.

The acidified tryptic peptide mixture was fractionated using SCX stage-tips equilibrated with 200 μL 0.2% trifluoracetic acid (TFA). The SCX stage tips were washed with 100 μL 1% TFA in isopropanol and subsequently with 100 μL 0.2% TFA in water. Peptides were eluted from the stage tips in three fractions by increasing pH and increasing organic solvent, first with 100 mM NH_4_HCO_2_, 40% ACN, 0.5% formic acid (FA), then 150 mM NH_4_HCO_2_, 60% ACN, 0.5% FA and finally with 5% ammonia, 80% ACN.^68^ Eluted peptides were vacuum dried and re-suspended in 6 μl 100 mM NH_4_HCO_2_, 40% ACN, 0.5% FA.

#### Plasma protein preparation

Fifty μl plasma was subjected to albumin and immunoglobulin depletion with the Albumin IgG removal kit (Merck). Fifteen μg of depleted sample was then loaded onto an SDS-PAGE gradient gel and after electrophoresis the gel lane was cut into 15 pieces. The gel pieces were subjected to in-gel digestion following a previously described protocol.^2^ In brief, the proteins were reduced with 10 mM DTT, alkylated with 55 mM IAA, and then incubated with trypsin overnight. The resulting peptides were collected, vacuum dried, and re-suspended in 0.1% formic acid.

#### Protein mass spectrometry data acquisition and analysis

Fractionated peptides in buffer A (0.1% FA) were loaded onto a 30-cm, 75-micron inner diameter column, packed in-house with ReproSil-Pur C18-AQ 1.9-micron beads (Dr. Maisch GmbH, Ammerbuch-Entringen, Germany) using the Easy-nLC 1200 autosampler (Thermo Fisher Scientific, Waltham MA, USA) at 60°C and Easy-nLC 1200 liquid chromatograph (Thermo Fisher Scientific). Separation was performed at 400 nl/min and applying a gradient of buffer B (80% ACN, 0.1% FA) from 2% to 30% over 120 min followed by a ramp to 60% over 10 min, to 95% over the next 5 min and keeping at 95% for another 5 min. Eluting peptides were directly sprayed into a Q Exactive HF benchtop Orbitrap mass spectrometer (Thermo Fisher Scientific). The mass spectrometer was operated in a data-dependent mode with survey scans from 300 to 1750 m/z, resolution of 60000 at m/z = 200, and up to 15 of the top precursors were selected and fragmented using high energy collisional dissociation with a normalized collision energy of 28. The MS2 spectra were recorded at a resolution of 15000 (at m/z = 200). AGC target for MS1 and MS2 scans were set at 3E6 and 1E5, respectively within a maximum injection time of 100 and 25 ms for MS and MS2 scans, respectively.

The mass spectrometry raw files were analyzed using Integrated Proteomics Pipeline (IP2) with ProLuCID, DTASelect2 and Census modules.^60^^,^^69^^,^^70^^,^^71^^,^^72^^,^^73^ The UniProt mouse protein database (download date: May 2021) was used. The tandem mass spectra were then matched to sequences using the ProLuCID algorithm with 20 ppm peptide mass tolerance for precursor ions and 50 ppm for fragment ions. To estimate peptide probabilities and FDRs accurately, target/decoy database was used containing the reversed sequences of all the proteins appended to the target database,^60^^,^^74^ and the FDR was controlled under 1% at peptide level. Each dataset was searched against both light (^14^N) and heavy (^15^N) protein databases. The results from ProLuCID were filtered using DTASelect2 and then were used to obtain quantitative ^14^N/^15^N ratios with Census software.^75^ For each protein, the comparison between the heavy and light forms was determined by considering the composite of all peptide ratios assigned to that protein. The protein composite ^14^N/^15^N ratio combines both data from singleton and non-singleton peptides in IP2. This approach is particularly beneficial for LLP data with numerous "singleton" data points. The ^14^N/^15^N (R) can be transformed to the "fractional abundance of old proteins" with the equation "R/(R+1)". Both values provide essentially the same information and are included in Table S1.

#### Tissue DNA preparation

DNA from 2 to 6 mg tissue (brain, thymus, heart, lung, liver, spleen, adrenal gland, kidney, pancreas, muscle) was isolated with the FitAmp General Tissue section DNA Isolation Kit (Epigentek, Farmingdale, NY, USA) and 500–1000 ng DNA subsequently digested into deoxynucleosides with the EpiQuik One-Step DNA Hydrolysis Kit (Epigentek) according to manufacturer’s instructions.

#### Deoxynucleoside mass spectrometry data acquisition and analysis

Deoxynucleoside samples (10–20 ng/μL) were diluted 1:10 with water and analyzed by high-performance liquid chromatography/mass spectrometry using a Nexera X2 liquid chromatograph (Shimadzu, Duisburg, Germany) with a Cortecs UPLC T3 column, 2.1 × 100 mm, 1.6 μm particle size (Waters, Eschborn, Germany) at a flow rate of 0.3 ml/min and 30°C, coupled to a QTrap 5500 triple quadrupole mass spectrometer (Sciex, Darmstadt, Germany).

Deoxynucleosides in 5 μl were eluted (eluent A: water/10 mM ammonium formate/0.1% FA, eluent B: methanol/10 mM ammonium formate/0.1% FA) using a 10 min gradient (0–0.5 min 5% B, 0.5–5 min 5-10% B, 5–5.5 min 10-95% B, 1 min held at 95% B, 6.5–7 min 95-5% B and 7–10 min 5% B). The ion source was operated in positive mode at 500°C, and multiple reaction monitoring (MRM) collision-induced dissociation (CID) was performed using nitrogen collision gas. The peak areas were integrated using Multiquant software (Sciex). The transitions and retention times monitored during analysis are shown in below table.

**Table:** Detection of deoxynucleosides by MRM

Compound	Q1 Mass	Q3 Mass	RT [min]	DP [V]	EP [V]	CE [V]	CXP [V]
Deoxyadenosine	252.0	136.0	4.8	56	10	23	12
^15^N-Deoxyadenosine	257.0	141.0	4.7	56	10	23	12
Deoxyguanosine	268.0	152.2	4.0	56	10	21	14
^15^N-Deoxyguanosine	273.0	157.2	4.0	56	10	21	14
Thymidine	243.0	127.0	4.8	61	10	21	12
^15^N-Thymidine	245.0	129.0	4.8	61	10	21	12
Deoxycytidine	228.0	111.9	1.6	56	10	27	12
^15^N-Deoxycytidine	231.0	114.9	1.5	56	10	27	12
Methyldeoxycytidine	242.0	126.1	2.3	46	10	21	10
^15^N-Methyldeoxycytidine	245.0	129.1	2.3	46	10	21	10

### Quantification and statistical analysis

#### Functional enrichment analyses

Gene Ontology (GO), KEGG pathway enrichment and protein-protein interaction (PPI) network analysis were performed by using Metascape (http://metascape.org/).^76^ A category with a *p* value <0.01 was considered as a significant enrichment. To generate the heatmaps, Tbtools^77^ and HiPlot^78^ platform were used. The disease enrichment was analyzed by Disgenet platform^79^ using R.

#### Weighted correlation network analysis

The coexpression network was constructed based on protein expression levels of mouse samples using the WGCNA package (version 1.70–3) in the R programming environment.^38^ We selected 2109 LLPs from all tissues to generate the scale-free network. To weight highly correlated genes, the soft thresholding power (β) was set as 8, and the minimal module size was set as 30. Modules with distance heights lower than 0.25 were merged, which resulted in 39 modules. To identify modules that are significantly associated with the measured organ or sex traits, expression profiles of each module were summarized by the module eigengene (ME) and the correlation between the module and the trait was calculated. The associations of individual genes with traits were quantified by Gene Significance (GS) value and for each module, module membership (MM) was defined as the correlation of the ME and the gene expression profile. Genes with both GS > 0.2 and MM > 0.8 were considered as hub genes. Moreover, several modules correspond to one tissue.

#### Statistical analysis

Statistical analyses were conducted using GraphPad Prism, v8 and R software.

## Supplemental information

Figure S1. Tissue-specific quantitative profiling of long-lived proteins, related to Figures 1 and 3

Figure S2. Supplemental information on WGCNA of long-lived proteins, related to Figure 4

Figure S3. Tissue-specific hub genes for long-lived proteins, related to Figure 4

Figure S4. Long-lived proteins from brain and disease, related to Figure 6

Table S1. Quantitative proteomics

Table S2. Widespread LLPs

Table S3. LLPs shared

Table S4. Hub genes of LLPs

Table S5. Quantification of deoxynucleotides
