# Supplementary material for: Long-lived proteins and DNA as candidate predictive biomarkers for tissue associated diseases
Source: iScience. 2024 Mar 28;27(4):109642. doi: 10.1016/j.isci.2024.109642 (PMC11022098; doi:10.1016/j.isci.2024.109642)
Supplement: Figure S1. Tissue-specific quantitative profiling of long-lived proteins, related to Figures 1 and 3 [file mmc1.pdf]

iScience, Volume *27*

Supplemental information

Long-lived proteins and DNA as candidate predictive biomarkers for tissue associated diseases

Xiaosong Liu, Bozidar Novak, Christian Namendorf, Barbara Steigenberger, Yaoyang Zhang, and Christoph W. Turck

Figure S1

A

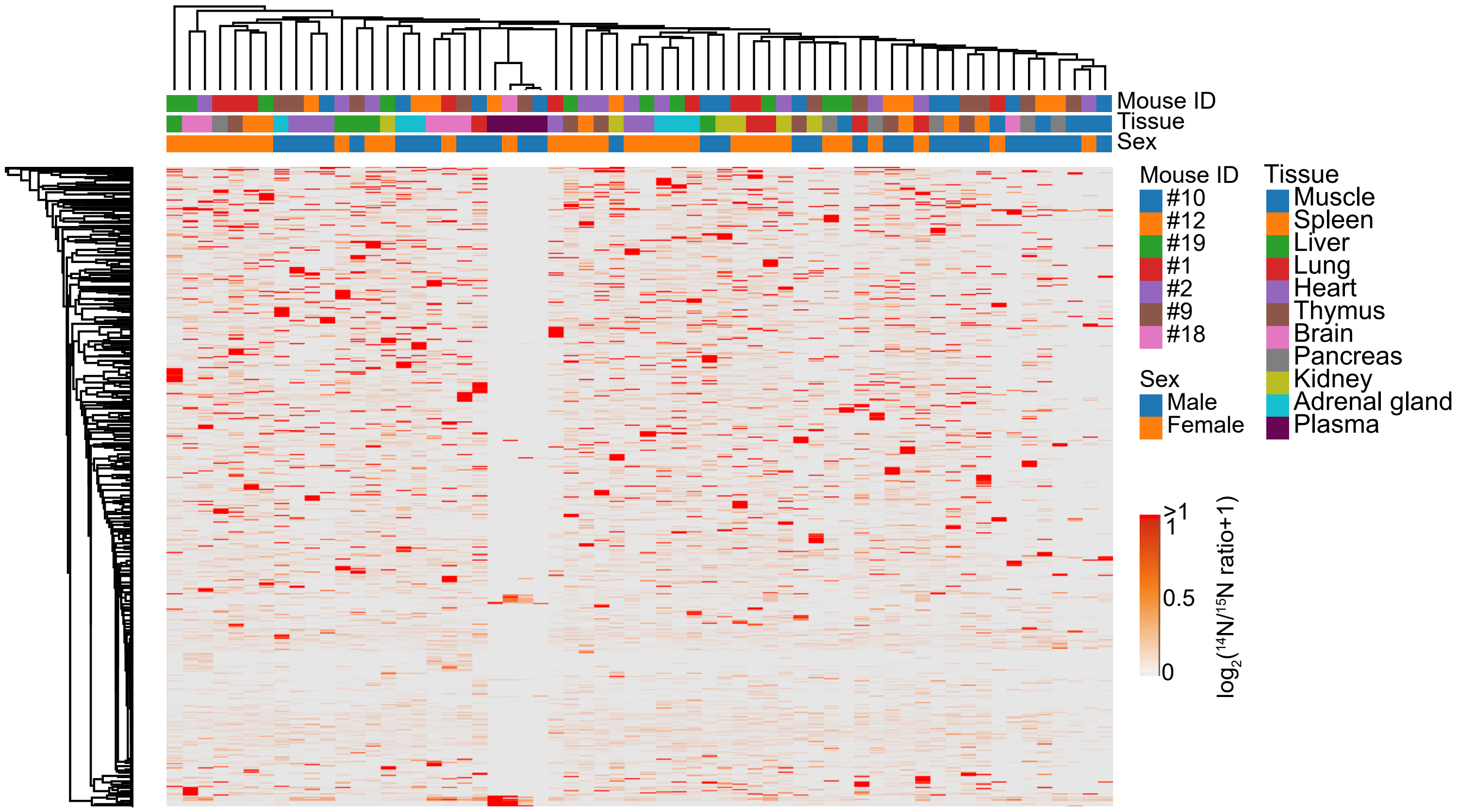

B

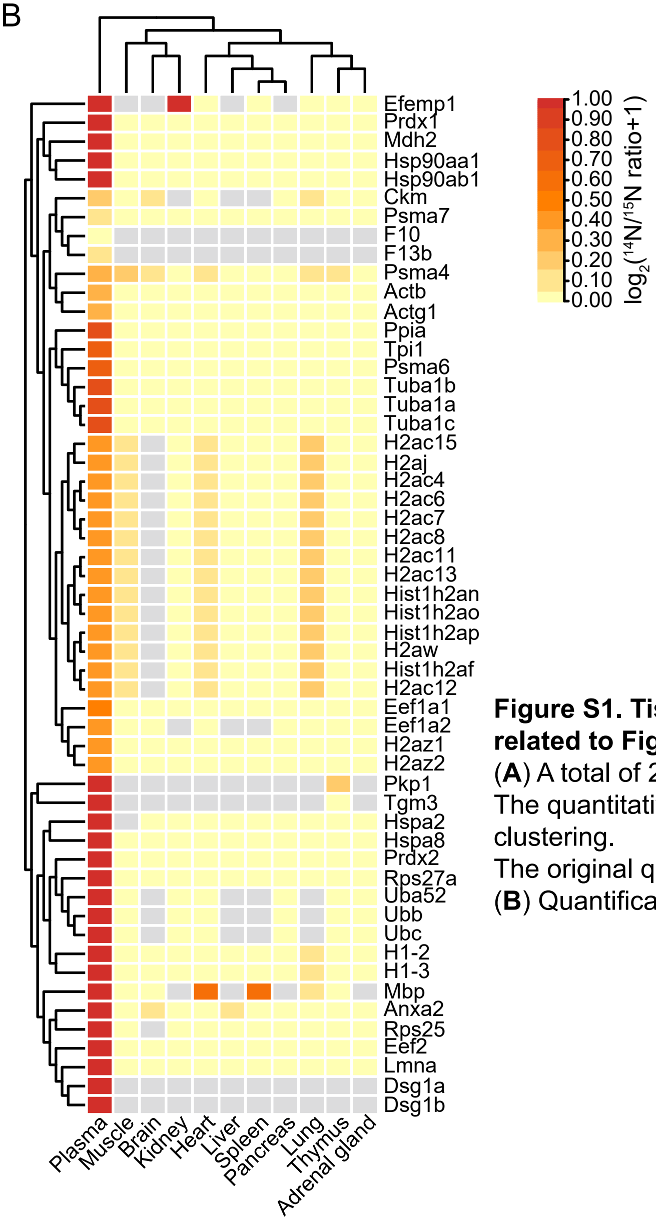

Figure S1. Tissue-specific quantitative profiling of long-lived proteins, related to Figure 1 and Figure 3.
(**A**) A total of 2113 long-lived proteins (LLPs) were quantified from all samples. The quantitative heat map was created by using average linkage hierarchical clustering.
The original quantitative values were standardized using $\log_2$ ($^{14}N/^{15}N+1$).
(**B**) Quantification of the 54 LLPs found only in plasma.
