# Supplementary figures and images for: Long-lived proteins and DNA as candidate predictive biomarkers for tissue associated diseases
Source: iScience. 2024 Mar 28;27(4):109642. doi: 10.1016/j.isci.2024.109642 (PMC11022098; doi:10.1016/j.isci.2024.109642)

Figure S2

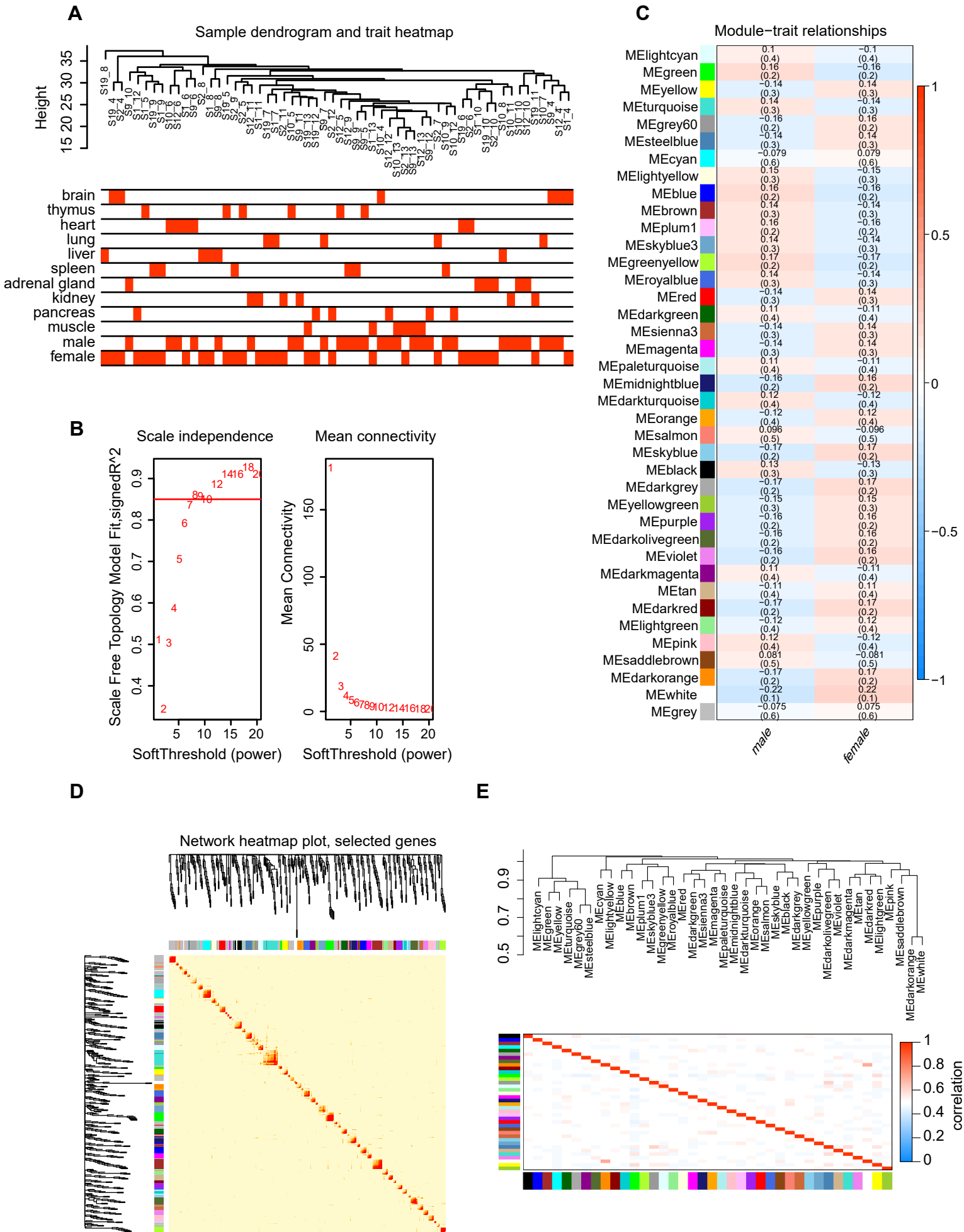

Supplement: Figure S2. Supplemental information on WGCNA of long-lived proteins, related to Figure 4 [file mmc2.pdf]
