# Supplementary material for: Long-lived proteins and DNA as candidate predictive biomarkers for tissue associated diseases
Source: iScience. 2024 Mar 28;27(4):109642. doi: 10.1016/j.isci.2024.109642 (PMC11022098; doi:10.1016/j.isci.2024.109642)
Supplement: Figure S3. Tissue-specific hub genes for long-lived proteins, related to Figure 4 [file mmc3.pdf]

Figure S3

A

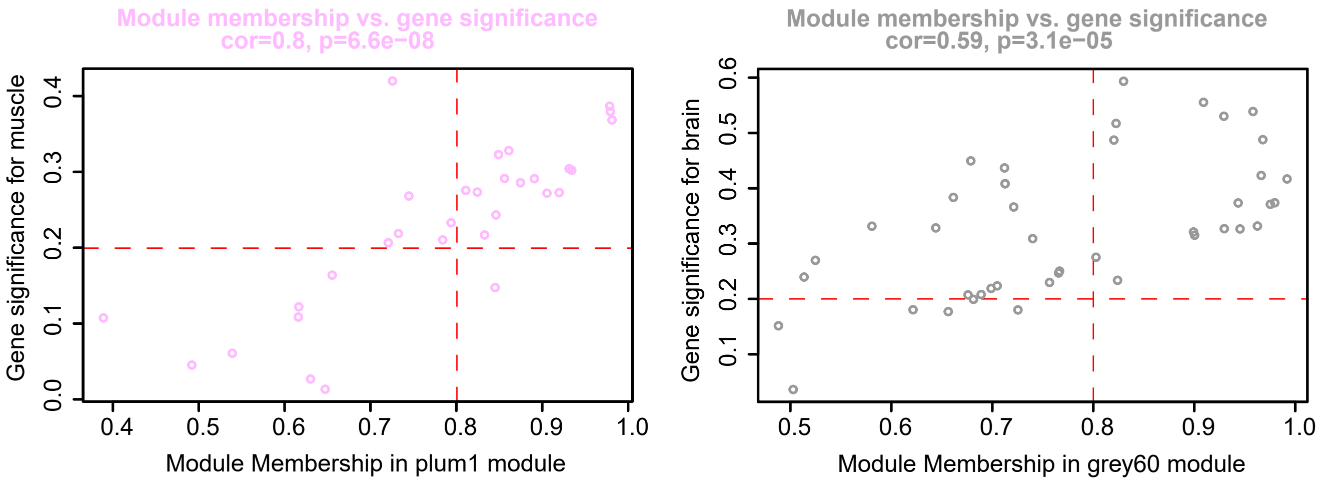

B

Table1. Tissues and modules (hub genes)

Tissue	Gene number	Module
Brain	152	green, grey60, lightcyan, steelblue, turquoise, yellow
Thymus	65	greenyellow, purple, skyblue
Heart	45	cyan, lightyellow
Lung	75	darkorange, pink, saddlebrown, white
Liver	107	darkgreen, magenta, paleturquoise, red, sienna3
Spleen	106	darkmagenta, darkred, lightgreen, royalblue, tan
Adrenal gland	63	black, brown, darkgrey
Kidney	101	blue, darkturquoise, midnightblue, orange, salmon
Pancreas	68	darkolivegreen, skyblue3, violet, yellowgreen
Muscle	17	plum1

Figure S2. Supplemental information on WGCNA of long-lived proteins, related to Figure 4.
(**A**) Clustering dendrogram of samples based on their Euclidean distance in WGCNA.
(**B**) Network topology for various soft-thresholding powers.
(**C**) Correlation between individual genes. Light color represents low overlap and dark red color represents high overlap.
(**D**) Correlation between modules. The module dendrogram is shown on top.
(**E**) Module–gender associations. Each row corresponds to a module, and each column represents a gender type.

Figure S3. Tissue-specific hub genes for long-lived proteins, related to Figure 4.
(**A**) In order to qualify as a hub gene for a module, the module is required to be positively correlated with a tissue type; gene significance should be greater than 0.2 and module membership should be greater than 0.8 in the WGCNA. Hub genes are represented in the upper right area in the plots.
(**B**) Hub genes and the corresponding modules in different tissues.
