# Supplementary material for: Long-lived proteins and DNA as candidate predictive biomarkers for tissue associated diseases
Source: iScience. 2024 Mar 28;27(4):109642. doi: 10.1016/j.isci.2024.109642 (PMC11022098; doi:10.1016/j.isci.2024.109642)
Supplement: Figure S4. Long-lived proteins from brain and disease, related to Figure 6 [file mmc4.pdf]

Figure S4

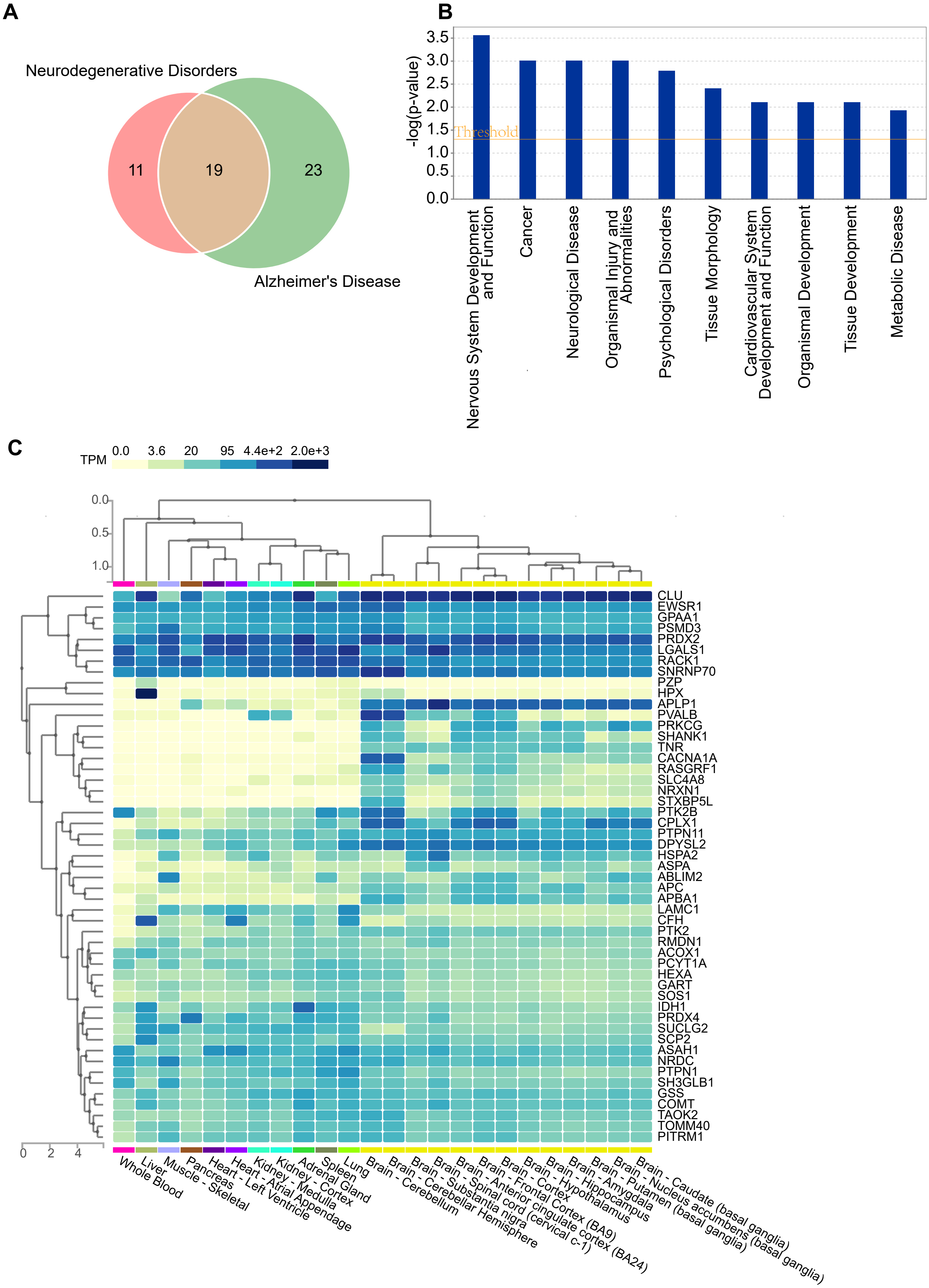

Figure S4. Long-lived proteins from brain and disease, related to Figure 6.
(**A**) Venn plot of long-lived proteins (LLPs) for Neurodegenerative Disorders and Alzheimer's Disease from Fig. 5C.
(**B**) Disease enrichment analysis using Ingenuity Pathway Analysis.
(**C**) Genes that are overexpressed in the brain.The analysis was performed using the GTEx platform and data from normal human tissues.
